# Hepatic Mitochondrial Function Analysis Using Needle Liver Biopsy Samples

**DOI:** 10.1371/journal.pone.0079097

**Published:** 2013-10-29

**Authors:** Michael J. J. Chu, Anthony R. J. Phillips, Alexander W. G. Hosking, Julia R. MacDonald, Adam S. J. R. Bartlett, Anthony J. R. Hickey

**Affiliations:** 1 Department of Surgery, University of Auckland, Auckland, New Zealand; 2 Applied Surgery and Metabolism Laboratory, School of Biological Sciences, University of Auckland, Auckland, New Zealand; 3 Maurice Wilkins Centre for Biodiscovery, University of Auckland, Auckland, New Zealand; 4 School of Medicine, University of Auckland, Auckland, New Zealand; 5 New Zealand Liver Transplant Unit, Auckland City Hospital, Auckland, New Zealand; CIMA. University of Navarra, Spain

## Abstract

**Backgrounds and Aim:**

Current assessment of pre-operative liver function relies upon biochemical blood tests and histology but these only indirectly measure liver function. Mitochondrial function (MF) analysis allows direct measurement of cellular metabolic function and may provide an additional index of hepatic health. Conventional MF analysis requires substantial tissue samples (>100 mg) obtained at open surgery. Here we report a method to assess MF using <3 mg of tissue obtained by a Tru-cut® biopsy needle making it suitable for percutaneous application.

**Methods:**

An 18G Bard® Max-core® biopsy instrument was used to collect samples. The optimal Tru-cut® sample weight, stability in ice-cold University of Wisconsin solution, reproducibility and protocol utility was initially evaluated in Wistar rat livers then confirmed in human samples. MF was measured in saponin-permeabilized samples using high-resolution respirometry.

**Results:**

The average mass of a single rat and human liver Tru-cut® biopsy was 5.60±0.30 and 5.16±0.15 mg, respectively (mean; standard error of mean). Two milligram of sample was found the lowest feasible mass for the MF assay. Tissue MF declined after 1 hour of cold storage. Six replicate measurements within rats and humans (n = 6 each) showed low coefficient of variation (<10%) in measurements of State-III respiration, electron transport chain (ETC) capacity and respiratory control ratio (RCR). Ischemic rat and human liver samples consistently showed lower State-III respiration, ETC capacity and RCR, compared to normal perfused liver samples.

**Conclusion:**

Consistent measurement of liver MF and detection of derangement in a disease state was successfully demonstrated using less than half the tissue from a single Tru-cut® biopsy. Using this technique outpatient assessment of liver MF is now feasible, providing a new assay for the evaluation of hepatic function.

## Introduction

Assessment of pre-operative liver function is an important selective process for identifying high-risk patients with chronic liver disease. Current methods include biochemical tests, imaging and histology [1]. The interpretation of biochemical (alanine and aspartate aminotransferase) tests remain subjective and relatively insensitive and histology-based assessments are strongly observer-dependant [2], [3]. Moreover, these tests do not provide direct information regarding hepatocyte metabolic function. One new approach to assess organ synthetic function is to directly measure mitochondrial function (MF). Mitochondria are responsible for the bulk of adenosine triphosphate (ATP) generation, the universal fuel source for cellular function [4]. Assessing MF therefore provides direct information of cellular energy homeostasis, and could provide important information in assessing hepatic function.

MF analyses test the oxidative phosphorylation (OXPHOS) and electron transport chain (ETC) systems, with an oxygraph device which measures oxygen flux. Titration protocols using selected substrates and inhibitors also permit the sequential measurement of flux through different respiratory mitochondrial complexes [5]. Conventional MF analysis has been performed in a research setting on isolated mitochondria [6], [7]. This method requires large tissue samples and as a result has precluded routine clinical mitochondrial research. Furthermore, the mitochondrial isolation process introduces bias through preferential selection of more healthy mitochondria. Technological advances using high-resolution respirometry have permitted down-scaling of sample requirements to approximately 10 mg of liver [6], [7]. Although significantly less than for isolated mitochondria, it still requires an open biopsy.

Percutaneous liver biopsy is within the scope of an outpatient procedure and is routinely performed for histological diagnosis [8]. If MF could be tested on similar needle biopsy samples, this could also allow for the development of a direct outpatient test of cellular metabolic function. To date there has been no published data using Tru-cut® needle biopsy samples for MF analysis. This study aimed to determine whether MF assays can be reliably performed on liver tissue obtained using Tru-cut® biopsies. The findings of this study will be relevant for enabling future new MF analysis in clinical hepatology.

## Materials and Methods

### Ethics statement

All animal experiments were conducted in accordance with the regulations provided by the Guide for Care and Use of Laboratory Animals, and were approved by the University of Auckland Animal Ethics Committee. The collection of human liver samples was approved by the New Zealand Northern Y Regional Ethics Committee and patients each gave written informed consent pre-operatively.

### Animal experiments

All reagents were purchased from Sigma-Aldrich (New South Wales, Australia) unless otherwise specified. Experiments were performed in male Wistar rats (350–450 g). The animals were kept under a 12-hour light/dark cycle (50–70% humidity, 22±2°C) with *ad libitum* access to standard chow (Teklad TB 2018; Harlan, Madison, WI) and water.

The rats were anaesthetized by isoflurane inhalation. A transverse laparotomy was performed. Tru-cut® samples were obtained with an 18G Bard® Max-core® biopsy instrument (Bard Biopsy Systems, Arizona, USA). The samples were immediately stored in ice-cold (∼4°C) University of Wisconsin (UW) solution (Madison, WI, USA).

### Human study

Tissue samples (27 patients; labelled in alphabetical order) were obtained from a normal parenchymal tissue site in the resected liver specimen using an 18G Bard® Max-core® biopsy instrument. The biopsy samples in all cases were immediately stored in ice-cold UW solution. Due to logistics of transport to the laboratory and processing the sample, our earliest time-point for MF analysis was 60 minutes.

### Optimal sample mass, stability and assay reproducibility

Tru-cut® specimens from both rats and patients were removed from ice-cold UW solution, bisected, blotted dry on lint-free lens tissue and weighed. Sample weights of 0.5, 1.0, 1.5 and 2.0 mg, and 0.5, 1.0, and 2.0 mg were used for rat and human MF analysis, respectively. Biopsy samples were randomized to each weight group prior to analysis. Three replicate measurements for each individual weight were obtained.

To test the stability of the Tru-cut® sample in cold storage, multiple Tru-cut® specimens were obtained from within the same rat (n = 4) or human (n = 3) livers, and stored in ice-cold UW solution. Rat liver MF was measured on matched but independently stored samples at 1, 2, 4, 8, 18 and 24 hours after each liver was sampled, while human liver MF was measured after 1, 2 and 4 hours. These time points were chosen to mimic various possible cold storage durations that a clinical Tru-cut® biopsy might realistically undergo prior to laboratory analysis in a hospital outpatient setting.

To test reproducibility, repeated Tru-cut® biopsies were obtained from rat (6 replicates; n = 6) and human livers (4 replicates; n = 6), and stored in ice-cold UW solution.

### Testing the utility of the assay to detect clinical changes in mitochondrial function

To test the ability of the Tru-cut® biopsy protocol to detect differences in MF, samples were obtained from ischemic rat liver samples and compared to matched perfused samples from the same rat liver. Six baseline perfused Tru-cut® samples were taken from rats (n = 6) and stored in ice-cold UW solution. Hepatic ischemia was then induced by 20 minutes of inflow occlusion (Pringle manoeuvre). After 20 minutes, six further biopsies were obtained from the same liver lobe and stored in ice-cold UW solution. Biopsy samples were analyzed immediately after procurement.

In a similar clinical study, recently resected human liver specimens were considered to be “ischemic” due to the effect of devascularization from the surgical resection process, whereas a matched biopsy from the remnant liver were considered “perfused”. Two Tru-cut® biopsies were obtained from both the resected and the remnant liver, and stored in ice-cold UW solution (n = 6).

### Evaluation of Tru-cut® biopsy against permeabilized wedge biopsy specimen

This experiment evaluated the Tru-cut® biopsy protocol against the current “gold standard” of using permeabilized wedge biopsy specimens. Two Tru-cut® biopsy specimens were obtained from patients undergoing liver resection (n = 6) and stored in ice-cold UW solution. Simultaneously, site-matched wedge biopsies of the same resected liver were taken using a 4 mm Stiefel (EBOS Healthcare, Auckland, New Zealand) and stored in ice-cold UW solution. Wedge biopsy specimens were removed from UW solution and mechanically permeabilized in ice-cold mitochondrial respiration media prior to analysis as per conventional protocols [7]. This study was only able to be undertaken on human clinical samples due to the fragile nature of the rat liver parenchyma which was unable to hold its structure in the vortex of stirred buffer in the oxygraph chamber.

### Respiration assays

Biopsy samples were removed from ice-cold UW solution, blotted dry on lint-free lens tissue and weighed. Due to the small and fragile nature of the Tru-cut® biopsy specimens, appropriately weighed samples were placed into the chambers of Oroboros Oxygraph 2K (Oroboros Instruments, Innsbruck, Austria) and saponin (50 µg/mL) was titrated directly into each chamber. This concentration was chosen as described by Kuznetsov et al [6]. This allowed direct permeabilization inside the chambers.

Respiration was measured in 2-mL chambers using the oxygraph, at 37°C in respiration media (final concentration in mM: 0.5 EGTA, 3 MgCl_2_, 60 K-lactobionate, 20 taurine, 10 KH_2_PO_4_, 110 sucrose and 1 mg/mL bovine serum albumin in 20 HEPES, pH 7.0 at 37°C), with a calculated saturated oxygen concentration of 190 nmol O_2_ per mL at 100 kPa barometric pressure. Weight-specific oxygen flux (pmol O_2_.s^−1^.mg wet wt^−1^) was calculated using the DatLab 5 analysis software. A simple substrate-inhibitor titration protocol was utilized to test the validity of the Tru-cut® biopsy protocol in assessment of Complex I (C-I) [5]. The assay was commenced with the addition of C-I supporting substrates: glutamate (10 mM), malate (5 mM) and pyruvate (10 mM) [LEAK_C-I_] followed by adenosine diphosphate (1.25 mM) [OXPHOS]. Atractyloside (0.25 mM) was added to inhibit the adenine nucleotide transporter (LEAK_Atra_). Incremental additions of carbonylcyanide *p*-trifluoromethoxy-phenylhydrazone (FCCP, final concentration 1.5 µM) were made to uncouple respiration as a measure of ETC capacity. Antimycin A (5 µM) was then added to inhibit Complex III and to provide a measure of residual oxygen flux. The integrity of tissue preparations and comparison of coupling efficiencies were made from the respiratory control ratio (RCR, OXPHOS/LEAK_C-I_). Assessment of C-I and RCR were chosen as it has been shown to be impaired in steatotic livers subjected to ischemia-reperfusion injury [9].

### Statistical and data analysis

All flux rate data from rat and human experiments are expressed as mean ± standard error of mean (SEM). The co-efficient of variation (CV) for each sample is expressed as the mean CV ± standard deviation (SD). Statistical analyses were performed using GraphPad Prism version 5.0 (GraphPad Software, San Diego, CA) for unpaired and paired t-tests. *P*<0.05 was considered statistically significant.

## Results

### Description of Tru-cut® biopsy specimen

**Figure 1:**
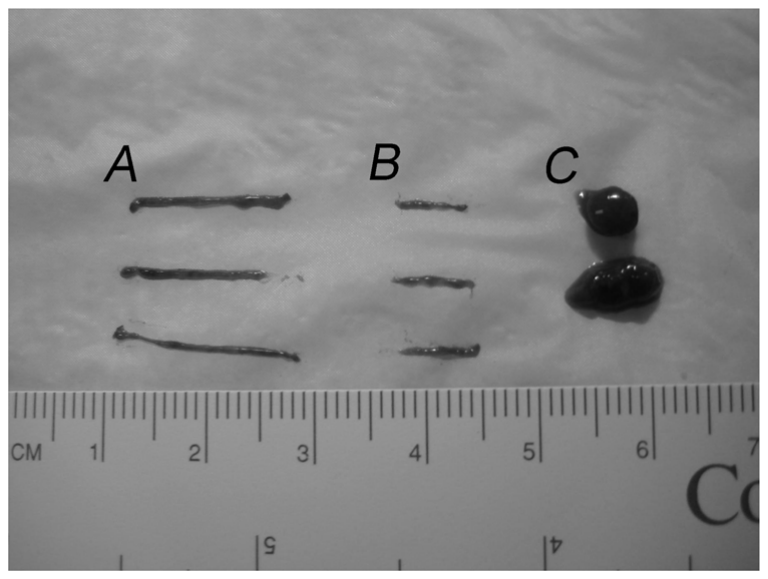
Sizes of Tru-cut® and wedge liver biopsies. (A) Intact Tru-cut® biopsy. (B) Transected Tru-cut® biopsy sample used for analysis. (C) Wedge biopsy sample.

Human biopsies were collected and bisected (Figure 1A, 1B), and were considerably smaller than the wedge biopsies (Figure 1C). The average mass of rat and human liver tissue obtained by a single Tru-cut® biopsy was 5.60±0.30 and 5.16±0.15 mg, respectively.

### Optimal liver sample mass for mitochondrial function analysis

The average mass of rat liver Tru-cut® samples used in each group was 0.66±0.10, 1.05±0.14, 1.56±0.12 and 2.13±0.21 mg. Sample masses of 1.5–2.0 mg had significantly lower mean CVs for OXPHOS, ETC capacity and RCR compared to 0.5 or 1.0 mg samples (Table 1). The mean flux rates were similar between each group. On this basis, the 1.5–2.0 mg sample size was used for all subsequent rat experiments.

**Table 1:** Mitochondrial function measurement across 4 different rat liver Tru-cut ®biopsy sizes (18 samples; 6 rats).

Sample mass	Mean ± SEM	Mean CV (%) ± SD^a^	CV (%)^b^
	**Oxidative phosphorylation (pmol O_2_.s^−1^.mg wet wt^−1^)**		
**0.5 mg**	8.1±0.9	39.1±26.1^c,d^	39.3
**1.0 mg**	9.5±1.2	35.6±15.6^c,d^	42.1
**1.5 mg**	9.6±0.7	12.9±8.4	26.5
**2.0 mg**	10.8±0.6	12.0±8.9	18.8
	**Electron Transport Chain (pmol O_2_.s^−1^.mg wet wt^−1^)**		
**0.5 mg**	18.5±1.3	18.7±11.0	24.1
**1.0 mg**	18.7±2.0	22.0±8.6^d^	36.2
**1.5 mg**	16.6±1.4	12.5±10.9	29.8
**2.0 mg**	15.2±0.8	8.8±9.6	18.8
	**Respiratory control ratio**		
**0.5 mg**	2.8±0.4	49.0±22.8^c,d^	54.7
**1.0 mg**	2.6±0.4	32.2±20.7^d^	54.0
**1.5 mg**	3.4±0.2	17.4±9.6^d^	22.9
**2.0 mg**	3.3±0.1	7.0±5.4	14.3

Oxidative phosphorylation is the measure of complex I State 3 respiration after the addition of complex I supporting substrates (10 mM glutamate, 5 mM malate, 10 mM pyruvate) and 1.25 mM ADP, while electron transport chain is the measure of the maximal electron transport chain capacity after the addition of the chemical uncoupler (carbonylcyanide *p*-trifluoromethoxy-phenylhydrazone, 1.5 µM FCCP). Respiratory control ratio is a measure of coupling efficiencies (rate in presence of ADP/rate in presence of complex I supporting substrates).

a , Mean CV from each of the 6 animals with calculated SD; ^b^, Combined CV of 18 replicates; Unpaired t-test: ^c^, *P*<0.05 compared to 1.5 mg; ^d^, *P*<0.05 compared to 2.0 mg. CV, Coefficient of variation; SD, Standard deviation; SEM, Standard error of mean

The average mass of Tru-cut® samples used from human patients was 0.61±0.03, 1.14±0.05 and 2.13±0.06 mg. A sample mass of 2.0 mg had lower mean CVs compared to 0.5 or 1.0 mg in measurements of MF (Table 2). The mean flux rates were comparable between each group. On this basis, 2.0 mg was used as the sample mass in subsequent experiments on human livers.

**Table 2:** Mitochondrial function measurement across 3 different human liver Tru-cut® biopsy mass (18 samples; 6 patients).

Sample mass	Mean ± SEM	Mean CV (%) ± SD^a^	CV (%)^b^
	**Oxidative phosphorylation (pmol O_2_.s^−1^.mg wet wt^−1^)** 1		
**0.5 mg**	6.3±0.9	39.1±32.6^c^	52.2
**1.0 mg**	5.7±0.5	23.5±14.7^c^	33.8
**2.0 mg**	7.4 ± 0.4	3.6±3.0	22.0
	**Electron Transport Chain (pmol O_2_.s^−1^.mg wet wt^−1^)** 1		
**0.5 mg**	9.0±1.1	36.1±16.2^c^	44.3
**1.0 mg**	7.4±0.7	21.4±19.0	36.1
**2.0 mg**	8.0±0.5	5.4±5.0	25.5
	**Respiratory control ratio** 1		
**0.5 mg**	3.1±1.1	43.2±40.9^c^	127.0
**1.0 mg**	3.2±0.9	26.2±29.8	97.9
**2.0 mg**	3.1±0.1	4.5±3.2	18.4

1 Refer to Table 1 legend for full definition.

a , Mean CV of 6 patients with calculated SD; ^b^, Combined CV of 18 replicates;

Unpaired t-test: ^c^, *P*<0.05 compared to 2.0 mg. CV, Coefficient of variation; SD, Standard deviation; SEM, Standard error of mean.

### Stability of Tru-cut® biopsy samples in cold storage

**Figure 2:**
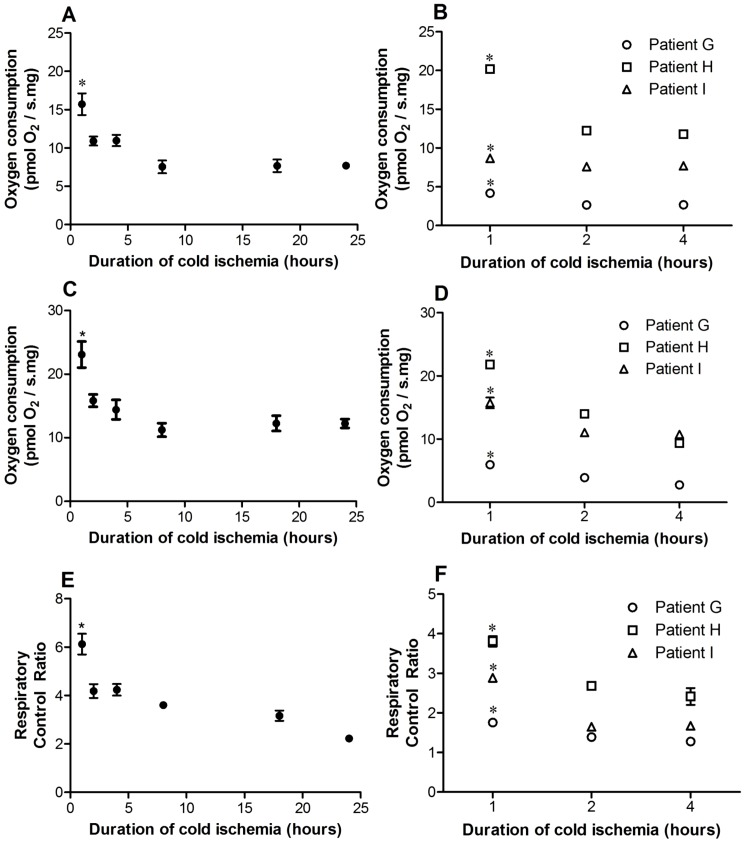
Mitochondrial function stability of rat and human Tru-cut® liver biopsies following different cold storage durations. Rat and human liver oxidative phosphorylation (A,B), electron transport chain capacity (C,D) and respiratory control ratio (E,F) at 1 hour was significantly higher compared to all other timepoints. Refer to the Table 1 legend for definition of oxidative phosphorylation, electron transport chain capacity and respiratory control ratio. Data are shown as mean ± standard error of mean (n = 3–4). *, *P*<0.05 (1 hour vs. other timepoints).

OXPHOS, ETC capacity and RCR of rat liver Tru-cut® samples following 60 minutes of cold storage were significantly higher compared to all other subsequent time-points (*P*<0.05, Figure 2A, 2C, 2E).

Human liver Tru-cut® sample OXPHOS, ETC capacity and RCR were also significantly higher following 60 minutes of cold storage compared to 2 and 4 hours of cold storage (*P*<0.05, Figure 2B, 2D, 2F). On the basis of these data, samples were only preserved up to 60 minutes in cold storage (this was the minimum possible time to undertake the transport and assay procedures).

### Precision: Mitochondrial function analysis was reproducible from repeated Tru-cut® sampling

**Figure 3:**
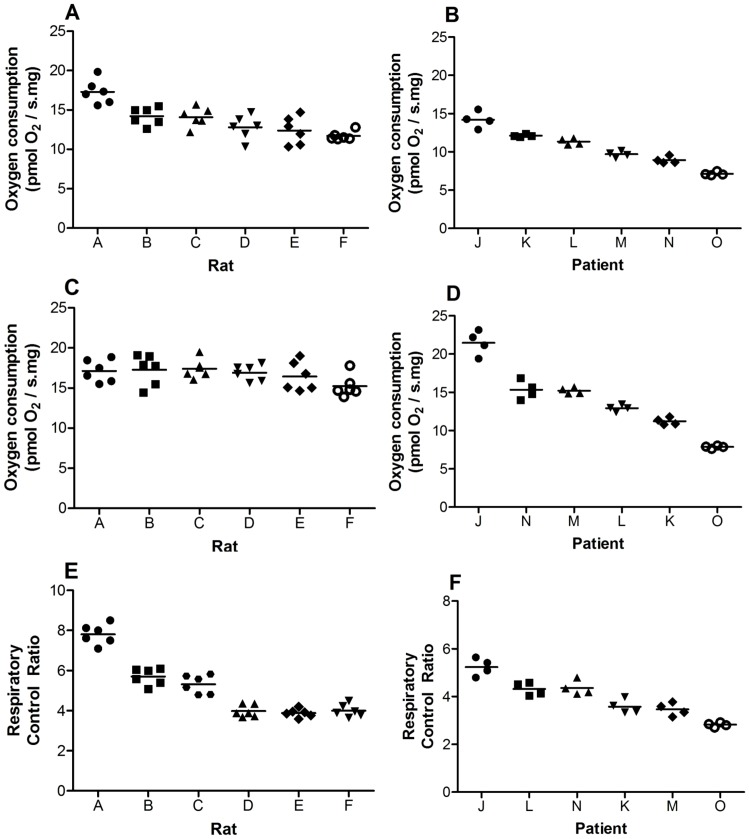
Reproducibility of rat (n = 6) and human (n = 6) Tru-cut® liver biopsies at 1 hour (2 mg). Oxidative phosphorylation (A,B), electron transport chain capacity (C,D) and respiratory control ratio (E,F) of individual replicates from rat and human liver are shown. Refer to the Table 1 legend for definition of oxidative phosphorylation, electron transport chain capacity and respiratory control ratio. The mean is shown as a horizontal bar. The data indicate that there is tight clustering of replicates around means.

The within individual OXPHOS, ETC capacity and RCR replicates from each rat liver is shown in Figure 3A, 3C and 3E, respectively. A tight cluster from each replicate was observed around their respective means. Consistent with this, the CV of the RCR was low from each rat (<10%). The mean OXPHOS, ETC capacity and RCR was different from each rat but this was expected due to inter-animal variability.

A similar finding was also observed from each human liver Tru-cut® biopsy (Figure 3B, 3D, 3F). A tight cluster was also observed around the mean of each patient and the CV was <10% from each patient.

### Utility: Tru-cut® sampling was able to detect a difference in mitochondrial function

**Figure 4:**
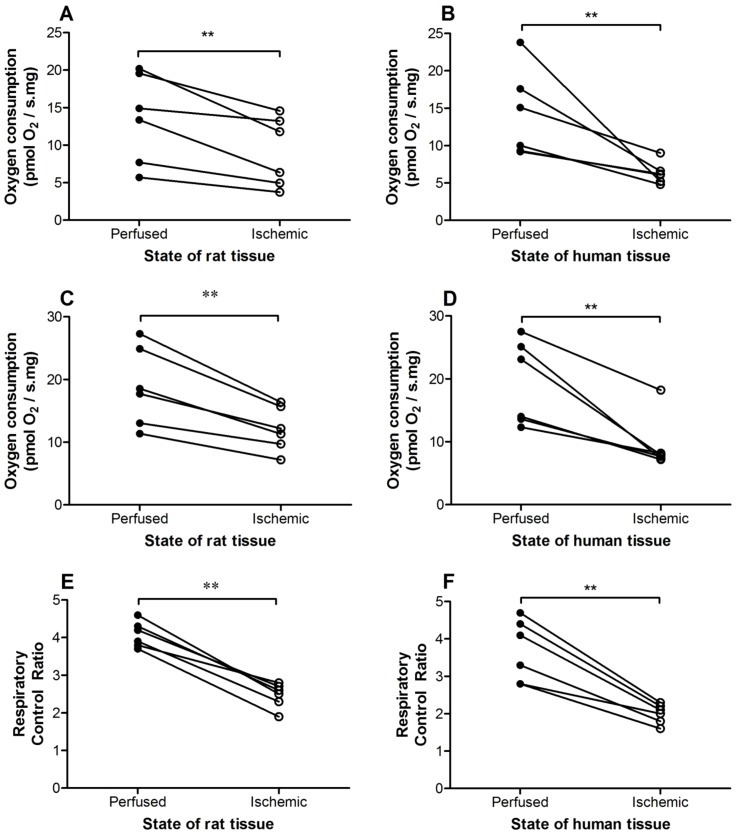
Utility of Tru-cut® biopsies from matched perfused and ischemic liver tissue samples. The duration of complete ischemia for rat and human liver biopsies were 20 and 12 minutes (range 6–16 minutes), respectively. Oxidative phosphorylation (A,B), electron transport chain capacity (C,D) and respiratory control ratio (E,F) of ischemic Tru-cut® rat (n = 6) and human (n = 6) liver samples were consistently lower than biopsies from perfused liver samples. Refer to the Table 1 legend for definition of oxidative phosphorylation, electron transport chain capacity and respiratory control ratio. **, *P*<0.01 vs. perfused samples.

Tru-cut® samples from ischemic rat livers consistently demonstrated lower OXPHOS, ETC capacity and RCR relative to the normally perfused samples from the same liver lobe (Figure 4A, 4C, 4E). These findings indicate damage to the ETC from the ischemic injury and that biopsies from Tru-cut® sampling were able to detect a difference in MF.

To evaluate a pathological MF change we used resected human liver samples. The average time from completion of the human liver resection to actual procurement of Tru-cut® samples from the resected specimen on the back-table was 12 minutes (range 6–16 minutes). This time in combination with the previous surgical ischemic stress induced during liver resection (median 99 minutes, range 70–120 minutes) was expected to be of sufficient duration to produce ischemic damage to the ETC. Although the difference in some cases was modest, OXPHOS, ETC capacity and RCR from resected human liver specimens were consistently lower compared to the remnant liver (Figure 4B, 4D, 4F).

### The Tru-cut® sample MF was equivalent to gold standard permeabilized wedge biopsy samples

There was no difference in the sample mass used in both groups (Tru-cut® biopsy: 2.75 ± 0.15 mg vs. permeabilized wedge biopsy: 2.75 ± 0.16 mg). OXPHOS, ETC capacity and RCR in Tru-cut® samples were similar to permeabilized wedge biopsy samples (Table 3). In some cases, Tru-cut® samples demonstrated significantly higher OXPHOS, ETC capacity and RCR (*P*<0.05). These findings suggest that the Tru-cut® sample was equivalent, and in some cases superior, to the current “gold” standard of tissue processing for MF analysis: permeabilized samples from a large wedge tissue sample.

**Table 3:** Comparison of Tru-cut® versus permeabilized wedge biopsy sample.

Patient	Mean flux ± SEM Tru-cut® biopsy	Mean flux ± SEM Permeabilized wedge biopsy
	**Oxidative phosphorylation (pmol O_2_.s^−1^.mg wet wt^−1^)** 1	
**V**	6.6 ± 0.3	7.4 ± 0.9
**W**	6.5 ± 0.4*	3.08 ± 0.1
**X**	23.7 ± 2.7*	10.0 ± 5.0
**Y**	6.4 ± 0.7	5.2 ± 0.5
**Z**	7.2 ± 2.6*	2.7 ± 0.4
**AA**	8.8 ± 0.9	8.9 ± 1.9
**Mean**	10.1 ± 1.9*	6.2 ± 1.1
	**Electron transport chain capacity (pmol O_2_.s^−1^.mg wet wt^−1^)** 1	
**V**	8.0 ± 0.4	9.4 ± 1.3
**W**	8.7 ± 0.4*	4.2 ± 0.6
**X**	23.5 ± 2.8*	12.3 ± 6.3
**Y**	10.4 ± 0.5*	7.0 ± 0.4
**Z**	9.4 ± 2.4	5.5 ± 1.2
**AA**	11.7 ± 0.1	9.3 ± 2.5
**Mean**	12.6 ± 1.6*	8.0 ± 1.2
	**Respiratory control ratio** 1	
**V**	2.0 ± 0.2	2.5 ± 0.3
**W**	2.3 ± 0.2*	1.5 ± 0.1
**X**	3.7 ± 0.5	3.4 ± 1.1
**Y**	5.5 ± 0.6*	2.7 ± 0.1
**Z**	2.3 ± 0.2*	1.5 ± 0.1
**AA**	3.1 ± 0.1*	2.5 ± 0.1
**Mean**	3.6 ± 0.4*	2.4 ± 0.3

1 Refer to Table 1 legend for full definition.

Paired t-test: *, *P*<0.05 vs. permeabilized samples; SEM, Standard error of mean.

## Discussion

In this study, we confirmed the feasibility of undertaking MF analysis on Tru-cut® liver biopsy samples. We demonstrated that it is possible to evaluate MF using as little as 2 mg of liver tissue, which is less than half the sample obtained by a standard percutaneous 18G Tru-cut® biopsy. The MF protocol was reproducible and able to detect differences in a range of samples. This new MF tissue analysis method was comparable to the current “gold” standard of a much larger liver wedge sample. To our knowledge, this is the first study to investigate the utility of MF in Tru-cut® biopsy samples from rodents and human patients.

Mitochondrial dysfunction has been implicated in a wide range of human pathologies including sepsis and multiple organ dysfunction syndrome [10]. MF has also been identified in hepatic steatosis as a key predictor of organ well-being [9]. MF analysis may provide a useful tool in the research of various liver diseases. Currently there are no detailed clinical studies of liver MF as there are no simple methods of procuring tissue for assessing MF. Here we show that MF analysis on very small biopsies can provide a new method for assessing hepatocyte function.

### Optimal sample size

A major barrier to routine clinical MF analysis is the large tissue requirement, making it necessary to obtain samples by open surgery [6], [7]. The development of high-resolution respirometry, combined with titration protocols, has provided a new method of standardized MF analysis [5]. This permits detection of small MF changes in small samples using a single assay employing a comprehensive sequential titration protocol [5]. This was the approach used for a pig livers study, where 30–40 mg, which was similar to an open wedge biopsy, was processed into 4–6 mechanically permeabilized subsamples of 2–7 mg prior to being successfully analysed [7]. A recent study has also shown that permeabilized liver samples had similar respiration rates as liver homogenate and indicated that there were no issues with diffusion in analyzing a permeabilized liver sample [11]. Furthermore, homogenization of such a small liver sample was found during our pilot investigation to be impractical with frequent and variable tissue loss to the homogenizer surfaces. This led us to conclude that a whole-tissue processing approach was the best method for reliably analyzing such a small sample size.

Our results suggest that MF analysis can be performed in 1.5–2.0 mg of rat liver Tru-cut® sample (Table 1). Consistent with the rat data, a human sample mass of 2.0 mg were also observed to have less variability than 0.5 or 1.0 mg (Table 2). This confirmed that despite differences in metabolic capacities between species, 2 mg of human liver Tru-cut® specimen is sufficient to measure MF. While this showed the capacity to detect oxygen flux in very small liver samples, the significantly higher CVs of <1.0 mg samples also indicate that there is greater variation in very small liver samples (0.5∼1.0 mg) (Table 1, 2). Importantly, these results indicate that only half of the Tru-cut® sample is required for MF analysis. This leaves the remaining tissue sample for standard histological analysis, which remains central to the description and diagnosis of liver diseases [8].

### Sample stability in cold storage

An important issue is the stability of the Tru-cut® sample in storage media, as this determines the urgency for analysis following sample procurement. If MF deteriorates rapidly, it would not be feasible to apply this protocol clinically due to the logistics required for rapid transport of the sample to a laboratory for analysis within a short timeframe. This may be facilitated by the presence of a high resolution oxygraph in the facility where liver biopsies are commonly performed. Our results indicate that OXPHOS, ETC capacity and RCR of rat and human liver Tru-cut® samples were compromized after 60 minutes of cold storage (Figure 2). These findings reflect the sensitivity of the electron transport C-I to cold ischemia [7]. One of the major roles of C-I is to pump protons across the inner mitochondrial membrane to assist in generating the proton gradient for ATP synthesis [10]. C-I dysfunction leads to decreased ATP synthesis and impaired cellular function (C-I flux contributes to approximately 2.5 ATP/oxygen molecule, Complex II approximately 1.5 ATP/oxygen molecule). However, the MF decline seen was faster than expected or previously reported by others [7]. This may be due to the small size of the samples analysed, increasing their sensitivity to cold ischemia. Our results suggest that Tru-cut® sample MF analysis should be performed within 60 minutes of procurement. This 60 minute timeframe would provide a suitable practical time limit for sample transfer to the laboratory and processing in preparation for analysis.

### Precision

The reproducibility or precision of a new method is an important factor to consider for further studies. Clinical diagnosis and patient management relies on data produced with minimal doubt of the precision. Our data indicate that Tru-cut® sampling was reproducible in analyzing MF with low CV and tight clustering of replicates around means of rat and human liver OXPHOS, ETC capacity and RCR (Figure 3). Four to six replicates were chosen as this provides sufficient number of repeats to determine precision but also minimize the time required to obtain the samples. Common laboratory tests were observed to have excellent precision (<10% CV) such that the main component to variation is biological variation [12], [13]. Our results suggest that the Tru-cut® protocol falls within these constraints.

### Utility

In addition to precision, a new diagnostic tool needs to be able to differentiate between “normal” and diseased states. Minor changes in cellular biology are often hard to detect, but small MF changes may indicate significant mitochondrial injury [5] and the ability to detect even subtle MF changes may improve therapeutic options. We used hepatic ischemia as a form of induced pathological injury to test our assay. Twenty minutes of ischemia generally delivers sufficient reversible injury [14], and we demonstrated that ischemic rat liver Tru-cut® samples consistently showed lower MF (Figure 4).

We also demonstrated that the Tru-cut® MF assessment protocol can also detect a difference in MF in humans. No deliberate hepatic ischemia was induced in this study but the progressive loss of perfusion during the resection process was used as a method of inducing ischemia. Additionally, the resected liver was left totally ischemic for an average of 12 minutes after completion of the resection before biopsies were taken. After this process we observed damage to the ETC. While this response may be variable (Figure 4) likely reflecting the variable time taken to resect the liver samples, resected liver specimen MF was consistently depressed. The RCR, which is independent of mass-specific flux, appears to provide a very consistent downward response to ischemia. Ischemic sample RCR were consistently lower than 2.5 (range 1.6–2.3) while perfused samples were consistently higher than 2.5 (range 2.8–4.7) (Figure 4F). This suggest that a RCR of <2.5 may potentially be “abnormal” but will need further clarification. Our findings confirmed the detection of ischemic ETC damage, and the potential utility of the Tru-cut® sampling to detect subtle MF changes.

### Accuracy

Wedge biopsies provide a large amount of tissue but are reliant on surgical resection. Needle biopsies can be performed as an outpatient, and appropriately-sized and placed needle biopsies have been shown to be superior to wedge biopsies for histological analysis [8]. Consequently needle biopsy sampling is routinely performed. The findings of our study open up the opportunity to add MF tests to the current biochemical and histological tests that are performed. While we employed a relatively simple protocol, it is also possible to undertake a more detailed assay array that includes assessing Complexes I, II, and IV; F_1_/F_0_-ATP Synthase and adenine nucleotide transporter [5]. Moreover advances in fluorimetry systems, which attach to the oxygraph, permit the measurement of ATP synthesis or reactive oxygen species [15]. Therefore there is a much greater capacity to gain considerable information beyond that presented here.

For optimal MF analysis, substrates (including oxygen) must not be limiting, unless particular affinities for these substrates are being sought. This requires permeabilization, homogenization or isolation in order to decrease diffusion distances [5]. Saponin or digitonin permeabilization of tissues has been routinely used for MF analysis [6]. Without adequate substrate access, the analysis would inaccurately represent the sample's mitochondrial population. The current method used for liver tissue permeabilization includes mechanical and/or chemical permeabilization of large liver samples to smaller samples [7]. We demonstrated that Tru-cut® samples were equivalent to permeabilized wedge biopsies in analyzing MF (Table 3). Overall we consider that the Tru-cut® sample was more reflective of the specimen in comparison to the wedge biopsy sample. Now that the conditions for sample collection have been developed, the use of standardized assay media, protocols and equipment; healthy baseline data in various clinical populations can be developed, which will provide a useful reference against which liver biopsies can be tested.

### Study limitation

One limitation is the potential error in weighing very small tissue samples, particularly samples of 0.5 mg. However, utilizing 2 mg of sample minimizes this error to an acceptable level. We used UW solution because it is the most common preservation solution used in liver transplantation and has been used to preserve donor liver grafts for up to 24 hours [16]. However, it would be worth investigating alternative preservation media (e.g. Histidine-tryptophan-ketoglutarate solution) as this may provide greater sample stability. It is standard convention to take 1 or 2 needle biopsy samples. There have been no previous studies on the association between liver anatomy and MF, and there may be some variation depending on where the biopsy is obtained. Further studies will be required to evaluate this relationship.

In conclusion, we demonstrated that Tru-cut® liver samples are suitable for routine MF analysis. The Tru-cut® biopsy protocol was reproducible, has the potential to detect subtle MF changes and was superior to permeabilized wedge biopsy samples. These findings suggest that percutaneous biopsies may have utility in the evaluation of liver bioenergetic status and offer additional information than is currently available with conventional biochemical and histological tests.
